# Suffering and Destitute

**Published:** 1867-09

**Authors:** 


### SUFFERING AND DESTITUTE.

The calamities of the war have fallen very heavily upon multitudes of innocent women and children in the once sunny South. Many of them have already starved to death in their lonesome cabins. On the 30th of July last an old subscriber, who lost ninety thousand dollars by the war, and a noble son on the field of battle, wrote thus: "Dr. HALL, the Southern people are now in the most humiliated condition possible; there is very little for us to wish to live for." In a previous letter he had stated that many families formerly rich and respectable, were in a state of absolute destitution, without means or money or available work.

Various means have been adopted by prominent Northern citizens, ladies and gentlemen, for the purpose of raising money to relieve present necessities, and to save multitudes from actual starvation, until opportunities are had for supporting themselves. One of these enterprises is in the shape of a "GRAND NATIONAL ENTERTAINMENT" for the aid of The Ladies' Society in aid of the suffering and destitute Poor of the South, to take place at Washington, September 30, 1867. The Managers are Bently, Clark & Co., 222 Pennsylvania Avenue, Washington, D. C. The Secretary of the Committee is Miss Harlow C. Mather; that Committee consists of Mrs. Laura Brooks, Mrs. Charles Wadsworth, Mrs. H. Sherman, Mrs. Drake Mills, Miss M. Duncan, Miss Mariah Moulton and Mrs. James Clark. Among the Honorary Members are Mrs. W. B. Astor, Mrs. A. T. Stewart, Mrs. August Belmont, Mrs. W. Aspinwall, Mrs. J. C. Fremont, Mrs. A. E. Burnside, Mrs. Governor Fenton, Mrs. C. N. Chapin, Mrs. L. W. Jerome and others. Names so eminent as these, we consider a guarantee of a good and honorable management. We trust that every fair and legitimate means will meet with a large reward, resulting in carrying welcome aid to thousands and thousands of deserving but suffering widows and orphans.

Single tickets are Two Dollars each, five for $9. 50 for $90. 100 for $180. Awards to ticket-holders vary in value from five dollars up to eighty thousand dollars. For tickets, or further information, apply to J. H. HALL, 102 Fourth Avenue, New York City.

